# Effects of six common dietary nutrients on murine intestinal organoid growth

**DOI:** 10.1371/journal.pone.0191517

**Published:** 2018-02-01

**Authors:** Tenson Cai, Yijun Qi, Albert Jergens, Michael Wannemuehler, Terrence A. Barrett, Qun Wang

**Affiliations:** 1 Department of Chemical and Biological Engineering, Iowa State University, Ames, Iowa, United States of America; 2 Biomedical Engineering Department, Case Western Reserve University, Cleveland, Ohio, United States of America; 3 Department of Veterinary Clinical Sciences, Iowa State University, Ames, Iowa, United States of America; 4 Department of Vet Microbiology and Preventive Medicine, Iowa State University, Ames, Iowa, United States of America; 5 Department of Internal Medicine, Division of Gastroenterology, University of Kentucky, Lexington, Kentucky, United States of America; University of Illinois at Chicago, UNITED STATES

## Abstract

The intestinal epithelium of the gastrointestinal (GI) tract constantly renews itself to absorb nutrients and provide protection for the body from the outside world. Since the intestinal epithelium is constantly exposed to various chemicals and dietary components, it is critical to determine which constituents promote or inhibit intestinal epithelium health and growth rate. Intestinal organoids, three-dimensional miniature models of the intestines, represent an ex vivo tool to investigate intestinal physiology and growth patterns. In this study, we measured the growth rates of murine intestinal organoids exposed to various concentrations of different dietary constituents. Results indicate that caffeic acid inhibited organoid growth in a concentration-dependent manner, curcumin exhibited variable effectiveness, and vitamin C had no effect on organoid growth.

## Introduction

A major function of the intestines is to absorb nutrients, liquids, and other substances into the body [1]. This process is made possible through the intestinal epithelium, which are a layer of specialized cells on the mucosa that act as a barrier to harmful substances. The epithelium is folded into many larger structures called villi to increase surface area and maximize absorption. Villi are composed of smaller absorptive units, termed microvilli, which increase the absorption surface further. Intestinal crypts reside at the bottom of the villi and contain stem cells, which replicate to produce progenitor absorptive cells, which mature as they migrate towards the villus tip. This is the process of intestinal epithelial renewal. These immature cells divide 4–5 times before differentiating into the four specialized cell types on the epithelium: absorptive enterocytes, mucous-secreting goblet cells, hormone-secreting enteroendocrine cells, and Paneth cells, which produce anti-bacterial peptides. Enterocytes, goblet cells, and enteroendocrine cells then migrate towards the villus tip, perform their functions, undergo apoptosis, and are removed from the body over a 3–5 day lifespan. Paneth cells, however, remain in the intestinal crypts where they participate in antibacterial defense [1]. Because the epithelia is exposed to different chemicals and substances, it is critical to determine how the epithelia responds to various dietary constituents that are ingested daily. In this study, we investigated the use of a murine intestinal organoid system as a model to study how various dietary constituents affect the growth rates of a functional gut (organoid) system.

Intestinal organoids are three-dimensional mini-models of the intestines with similar anatomical features [2–3]. Intestinal organoids can be grown from single Lgr5-CBC cells, which are multipotent stem cells [3]. Because intestinal organoids resemble a functional gut system, they serve as effective models to study stem cell physiology, drug delivery, epithelial functions, and other processes. Moreover, organoids can be observed in real time [4] and may serve as effective disease models for debilitating disorders including inflammatory bowel disease (IBD) and GI cancer [3] and toxins such as deoxynivalenol [5]. Cancer models have been generated to observe the way the gut behaves during malignancy. Intestinal epithelial cell physiologies and functions have been studied in animals such as piglets [6–8]. There is also potential for regenerative therapy where damaged epithelial cells can be replaced with organoids cultivated ex vivo [3, 9].

Caffeic acid is a phenolic compound found in plants [10]. Chlorogenic acid is an ester formed from caffeic acid and quinic acid [11] that is found in coffee [10]. These phenolic compounds are antioxidants that may help prevent cardiovascular diseases [10], such as atherosclerosis, and cancer development. Chlorogenic acid has also been reported to prevent DNA damage by monochloramine, which may help prevent gastric mucosal lesions [12]. Caffeic acid inhibits cancer cell proliferation [13]. Olthof et al. [10] conducted a study on the absorption of chlorogenic acid and caffeic acid in humans. They found that 33% of the ingested chlorogenic acid and almost all the ingested caffeic acid are absorbed by the small intestines. Therefore, it is important to study how growth and maturation of intestinal stem cells are influenced caffeic acid and chlorogenic acid.

L-Glutamic acid monosodium salt hydrate (MSG) is a commonly used food additive in Asian countries. Feng et al. [14] studied the effects of MSG and dietary fat on the health of pig intestinal systems and concluded that MSG decreased duodenal crypt depth. They also reported changes in intestinal morphology in response to the administration of probiotics and the effects of pathogen populations in the intestines. L-Ascorbic acid, or vitamin C, is a nutrient that is essential for host survival and is commonly found in fruits and vegetables. Vitamin C provides multiple benefits, including the promotion of wound healing since it increases collagen synthesis [15]. Vitamin C is also needed for the body to make muscle carnitine [16]. The liver requires this vitamin for the transformation of cholesterol into bile acids. This serves to reduce cholesterol buildup that can promote hypercholesterolemia, cholesterol gallstones, and other pathological conditions [17]. Still other, many reported health benefits of vitamin C, such as prevention of the common cold and other diseases including cancer, are still under investigation [15].

Curcumin is an antioxidant found in the plant *Curcuma longa* that acts as an anti-inflammatory, antioxidant, and antitumor agent [18]. There have been numerous studies on curcumin and its anticancer properties. Kakarala et al. [19] first reported that curcumin, along with another molecule called piperine, inhibits breast cancer stem cell renewal by inhibiting the Wnt signaling pathway. In another study, Dhandapani et al. [20] found that curcumin prevented glioblastoma cancer stem cell growth and chemoresistance by regulating the activity of transcription factors. Curcumin does not negatively affect normal cells [21], but how curcumin affects stem cells is currently unknown.

m-hydroxyphenylpropionic acid (mHPP) is a product produced from the catabolism of caffeic acid by the gut microbiota [22]. There are only a few studies reporting the physiologic effects of mHPP in mammalian hosts. Konishi and Kobayashi [22] conducted a study on the absorption of mHPP through the monocarboxylic acid transporter in Caco-2 cells.

The purpose of the current study was to determine the effects of different dietary constituents on intestinal organoid growth. Specifically, the sizes and growth rates of the organoids following exposures to the dietary constituents were examined by measuring and graphing the surface areas.

## Materials and methods

### Materials

All dietary constituents (caffeic acid, chlorogenic acid, MSG, vitamin C, curcumin, and mHPP) were purchased from Sigma-Aldrich. Matrigel was purchased from Corning Inc. Growth factors (EGF, Noggin, and R-spondin-1) were purchased from PeproTech Inc. The remaining materials were purchased from Life Technologies unless indicated otherwise.

### Isolation of intestinal stem cells (ISCs)

The protocol for isolating intestinal stem cells and preparing the ISC culture solution from Peng et al. [23–24] was followed. The intestinal crypts along with the intestinal stem cells were obtained from the proximal half of the mouse small intestine. C57BL/6 (B6) mice donated by the College of Veterinary Medicine of Iowa State University were used. All animal procedures were conducted with the approval of the Iowa State University Institutional Animal Care and Use Committee. All methods and procedures in the experiments were performed in full compliance with the Committee’s guidelines and regulations.

CO_2_ was used to anesthetize the mice and sacrifice was performed on the mice. The mice were cut open longitudinally and ice-cold PBS was used to remove most of the intestinal contents. Pieces of intestinal tissue (2–4 mm) were excised using scissors and placed into a 50 mL Falcon tube. Then, 30 mL of ice-cold PBS was added to the tube, and a 10 mL pipette was used to wash the intestinal tissue pieces. The supernatant was collected and discarded once the tissue pieces had descended to the bottom of the tube. This washing procedure was performed 5–10 times until the supernatant was mostly clear. Ice-cold 2 mM EDTA PBS buffer (30 mL) was then added to the tube. The tube was lightly rocked at 4°C for 30 minutes. The supernatant was discarded once the intestinal pieces settled.

Next, 20 mL of ice-cold PBS was added to the Falcon tube, and a 10 mL pipette was used again to wash the pieces up and down. Once the fragments had settled to the bottom, inverted microscopy was used to observe if the supernatant contained many villi or crypts. This step was repeated until most crypts were liberated. The crypt fragments were filtered through a 70 μm cell strainer (Corning Inc.) and gathered into a BSA-coated 50 mL Falcon tube. The leftover fragments on the strainer were discarded. The crypt pieces were centrifuged at 300 × *g* for 5 minutes. The cell pellet that formed was washed in a 10 mL ice-cold basal culture medium and placed into a BSA-coated 15 mL Falcon tube. The tube was centrifuged at 150 × *g* for 2 minutes to eliminate any individual cells. The crypts were washed and centrifuged 2–3 times until most of the individual cells were removed. An inverted microscope was then used to count the number of crypts. Intestinal crypts were suspended in an ice-cold basal culture medium and placed on ice.

### Preparation of ISC culture solution

The basal culture medium was made with Advanced DMEM/F12 along with 2 mM GlutaMax, 10 mM HEPES, and 100 U mL^-1^ penicillin/100 μg mL^-1^ streptomycin. This medium was then homogenously mixed with N2 supplement (1X), B27 supplement (1X), 1 mM *N*-acetylcysteine, 1 μg mL^-1^ R-spondin-1, 100 ng mL^-1^ Noggin, and 50 ng mL^-1^ EGF to form the final ISC culture medium.

After isolating crypts and preparing the culture medium, 1 × 10^4^ individual crypts were re-suspended in 1 mL Matrigel. Matrigel containing crypts (200 μL) was applied to two pre-warmed 24-well Falcon plates (Corning Inc.). The gel was deposited in the center of the well, and the plates were placed in an incubator at 37°C for 30 minutes for gelation to occur. Finally, 500 μL of ISC culture medium was added to each well. The plates and cells were maintained in a 37°C CO_2_ incubator.

### Dietary constituent preparation and treatment

**Fig 1:**
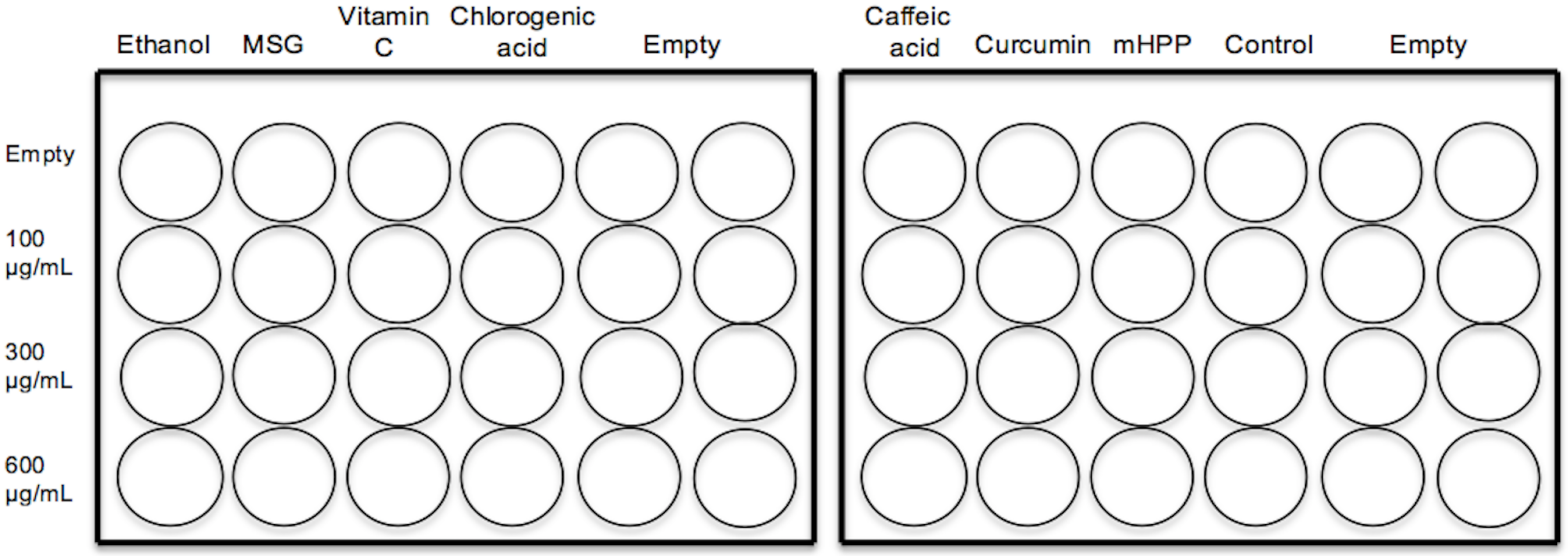
Experimental setup of dietary constituents. Dietary constituents were added in different concentrations (100 μg/mL, 300 μg/mL, and 600 μg/mL) to two 24-well plates. No constituents were added for the control group. Three organoids were chosen from each well to be monitored throughout the experiment.

Each dietary constituent was prepared into solutions of 100 μg/mL, 300 μg/mL, and 600 μg/mL. After growing the organoids for 7 days, cells were treated with dietary constituent solutions on day 8 (Fig 1). The dietary constituents were dissolved using different methods because they had different saturation points, and these methods are described in S1 Table. Because curcumin was dissolved in ethanol, a group of organoids was given only ethanol as a vehicle control. Another group of organoids that did not receive dietary constituents served as a control.

### Microscopy methods and organoid observation

**Fig 2:**
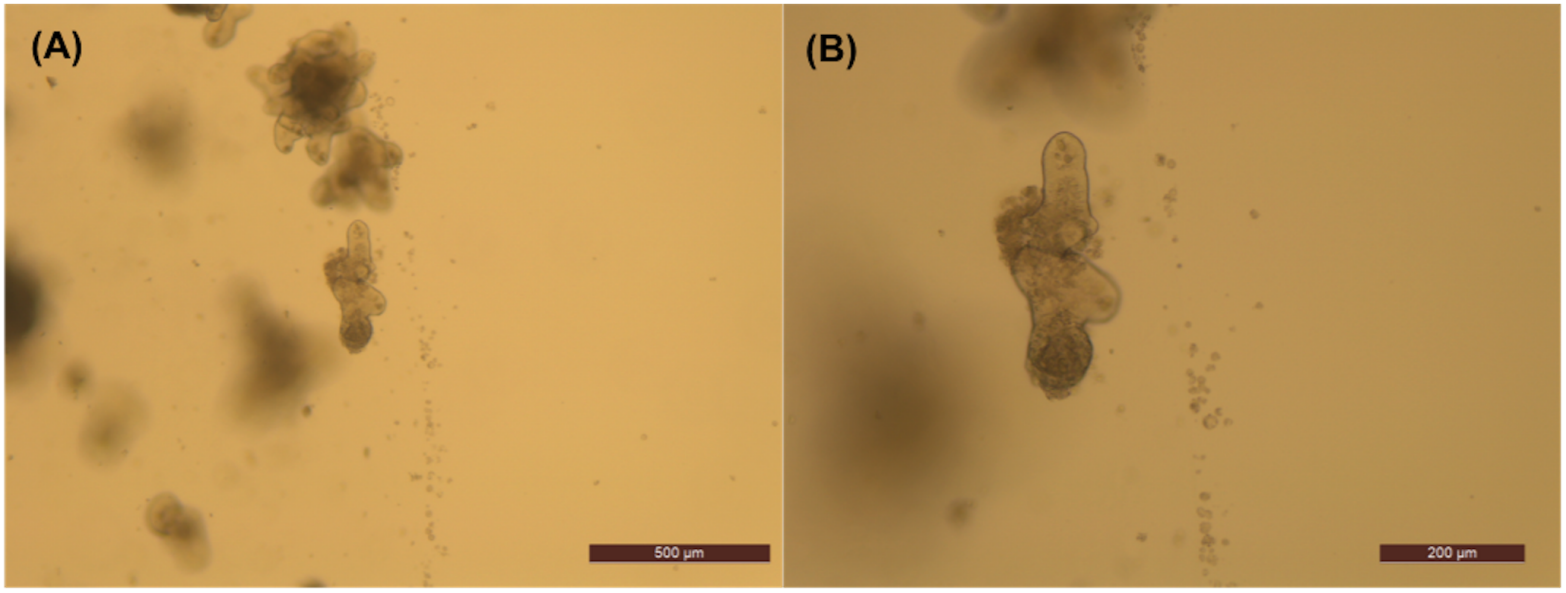
Images of murine small intestinal organoids composed of crypts and stem cells. (A) A 5X magnification image of an organoid. Scale bar: 500 μm. (B) A 10X magnification image of an organoid. Scale bar: 200 μm.

After administering dietary constituents on day 8, organoid growth was monitored daily for 8 days. The organoids were grown and monitored for a total of 15 days. Pictures of representative organoids were taken daily with the Leica Application Suite (LAS) software at 5X and 10X magnifications (Fig 2). These magnifications were suitable because 5X magnification provided a representative view of the organoid population in each well, while 10X magnification provided greater detail of individual organoids.

### Statistical analysis

After 15 days, ImageJ software was used to calculate the surface area of each representative organoid. The surface area was calculated only in the expanding horizontal directions. The vertical direction of expansion was not included in the calculations. Surface areas of representative organoids in each well were averaged and graphed. The surface area growth expansion rates were also calculated for each selected organoid in each well. The expansion rates were calculated with the formula (*Present Day Area* / First Day Area) * 100.

The standard deviations were also calculated and included. An unpaired, two-tailed Student’s t-test was performed on the expansion rate datasets to determine statistical differences between the dietary constituent-exposed organoids and the control group. An alpha value of 0.05 was used as the threshold for statistical significance in the t-test. Asterisks on the expansion rate graphs indicate P values that are less than 0.05.

## Results

The dietary constituents were added to the organoids on day 8 for all conditions. All pictures were taken with LAS software. Surface areas were calculated using ImageJ. PCR and western blot techniques were not utilized in this study.

### Monosodium glutamate

**Fig 3:**
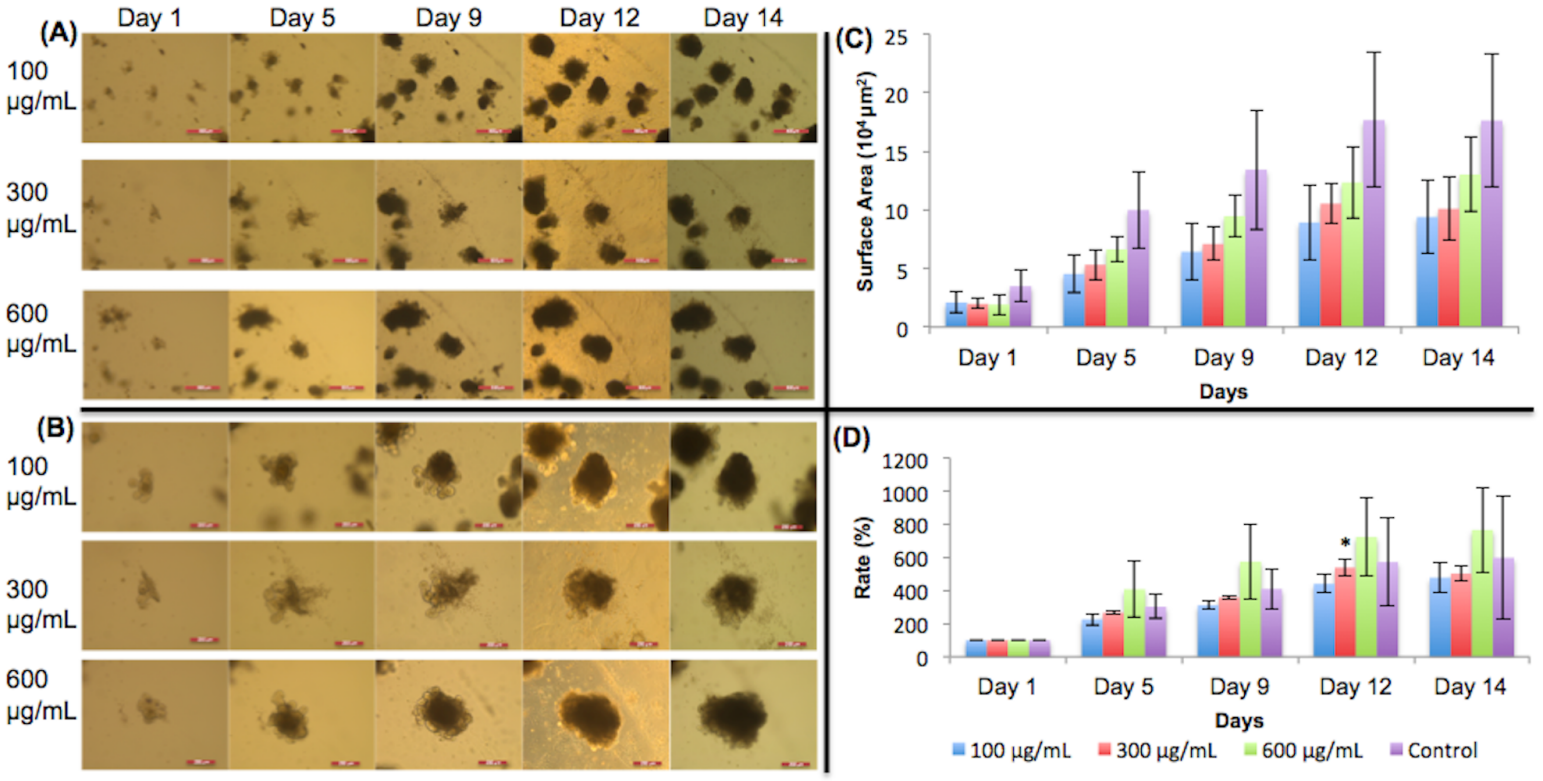
Monosodium glutamate treated organoid growth patterns. The surface areas of three organoids from each concentration of monosodium glutamate were measured. (A) 5X magnification of organoids given monosodium glutamate. Day 9 –day 14 show organoids with added dietary constituent. Scale bar: 500 μm. (B) 10X magnification. Day 9 –day 14 show organoids with added dietary constituent. Scale bar: 200 μm. The crypts were still fully visible on day 14, meaning there were fully functional stem cells and that monosodium glutamate did not inhibit organoid growth. (C) Surface area of organoids. (D) The surface area expansion rates of organoids were determined by the formula: (Present Day Surface Area)/(First Day Surface Area) * 100%. Significant growth occurred in 300 μg/mL on day 12. Examining the error bars, lower doses of MSG (100 and 300 μg/mL) reduced the variability (smaller error bars) in the surface area expansion rates compared to the 600 μg/mL and the control. Significance values were calculated using unpaired, two-tailed Student’s t-test with n = 3. *p<0.05.

Fig 3A shows 5X images of organoids treated with different concentrations of ethanol and their growth progression over time. Fig 3B shows the 10X images of the organoids. In both (A) and (B), the organoids chosen to be monitored are shown in the center of the image. The surface areas of the organoids are shown in Fig 3C. Some standard deviations were large due to large variation in organoid size. Expansion rates are shown in Fig 3D. The expansion rates were all 100% on day 1 because the organoids had not made any changes in growth. Organoids did not show the expected pattern of growth when MSG was added (Fig 3D). A previous study by Feng et al. [14] found that MSG inhibited crypt growth. However, as seen in Fig 3B, the crypts of the organoids that contain stem cells were still fully visible on day 14, meaning that cell differentiation and organoid growth were still occurring. Fig 3D supports this observation graphically. The expansion rates of the organoids given MSG did not indicate significant growth inhibition. On day 12, the 300 μg/mL organoids showed significant growth change from the control group (p<0.05). However, the growth change was not continued out to day 14.

### Vitamin C

**Fig 4:**
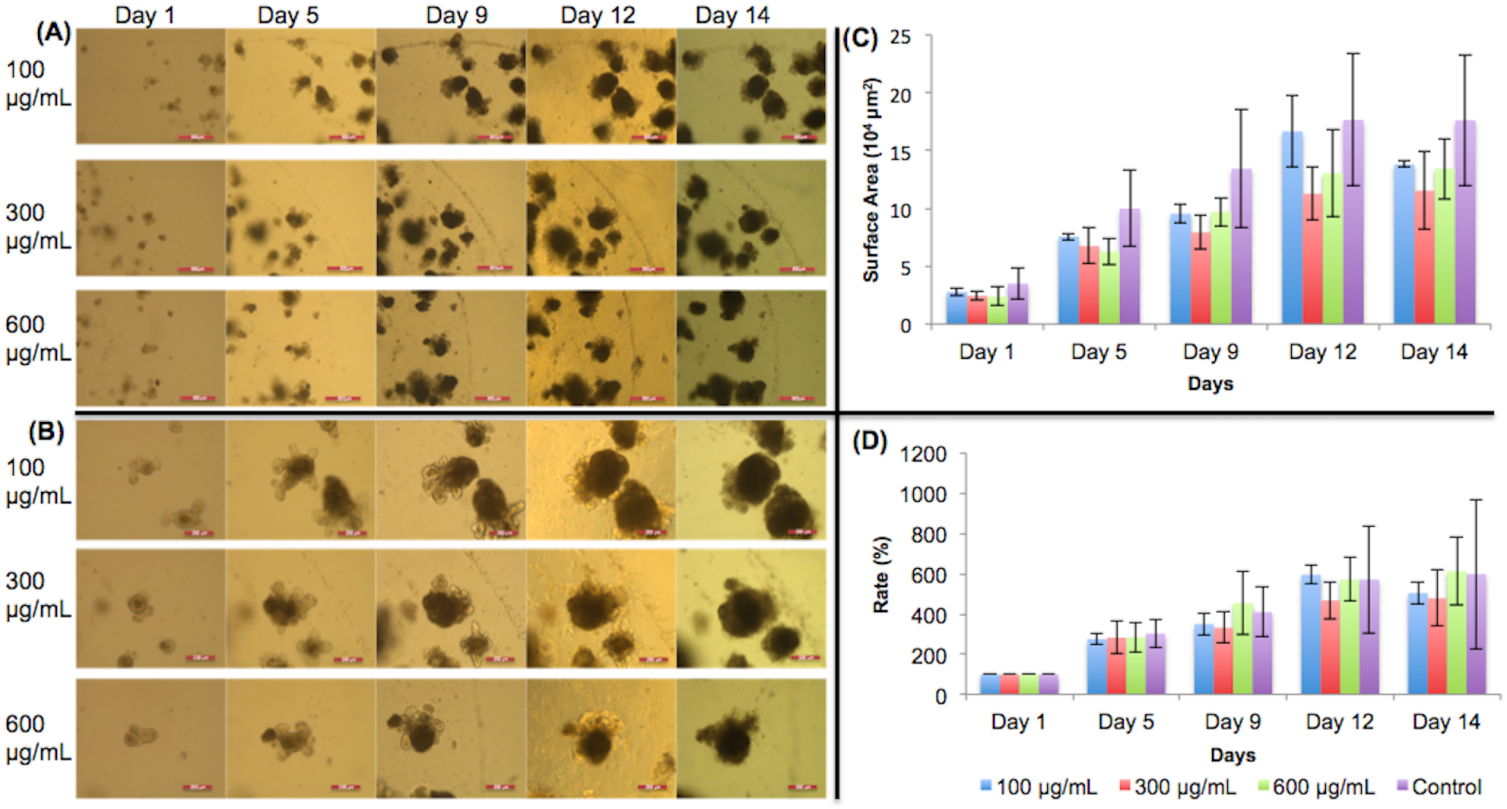
Vitamin C treated organoid growth patterns. The surface areas of three organoids were measured for each concentration in each day. (A) 5X magnification of organoids given vitamin C. Day 9 –day 14 show organoids with added vitamin C. Scale bar: 500 μm. (B) 10X magnification. Day 9 –day 14 show organoids with added vitamin C. Scale bar: 200 μm. (C) Surface area of organoids. (D) Surface area expansion rate of organoids. All treated organoids grew similarly to the control. No significant growth changes occurred. Significance values were calculated using unpaired, two-tailed Student’s t-test with n = 3. *p<0.05.

Fig 4A and 4B show 5X and 10X images, respectively, of organoids treated with different concentrations of vitamin C and their growth progress over time. The organoid surface areas calculated are shown in Fig 4C. In Fig 4D, organoids on days 1 and 5 showed approximately the same expansion rates among all groups, which is to be expected because vitamin C had not been added yet. There was more variation in growth after day 8, in the presence of the vitamin. However, all vitamin C-treated organoids grew similarly to the control and did not significantly differ from the control (p>0.05). We conclude that vitamin C did not significantly affect intestinal organoid growth.

### Chlorogenic acid

**Fig 5:**
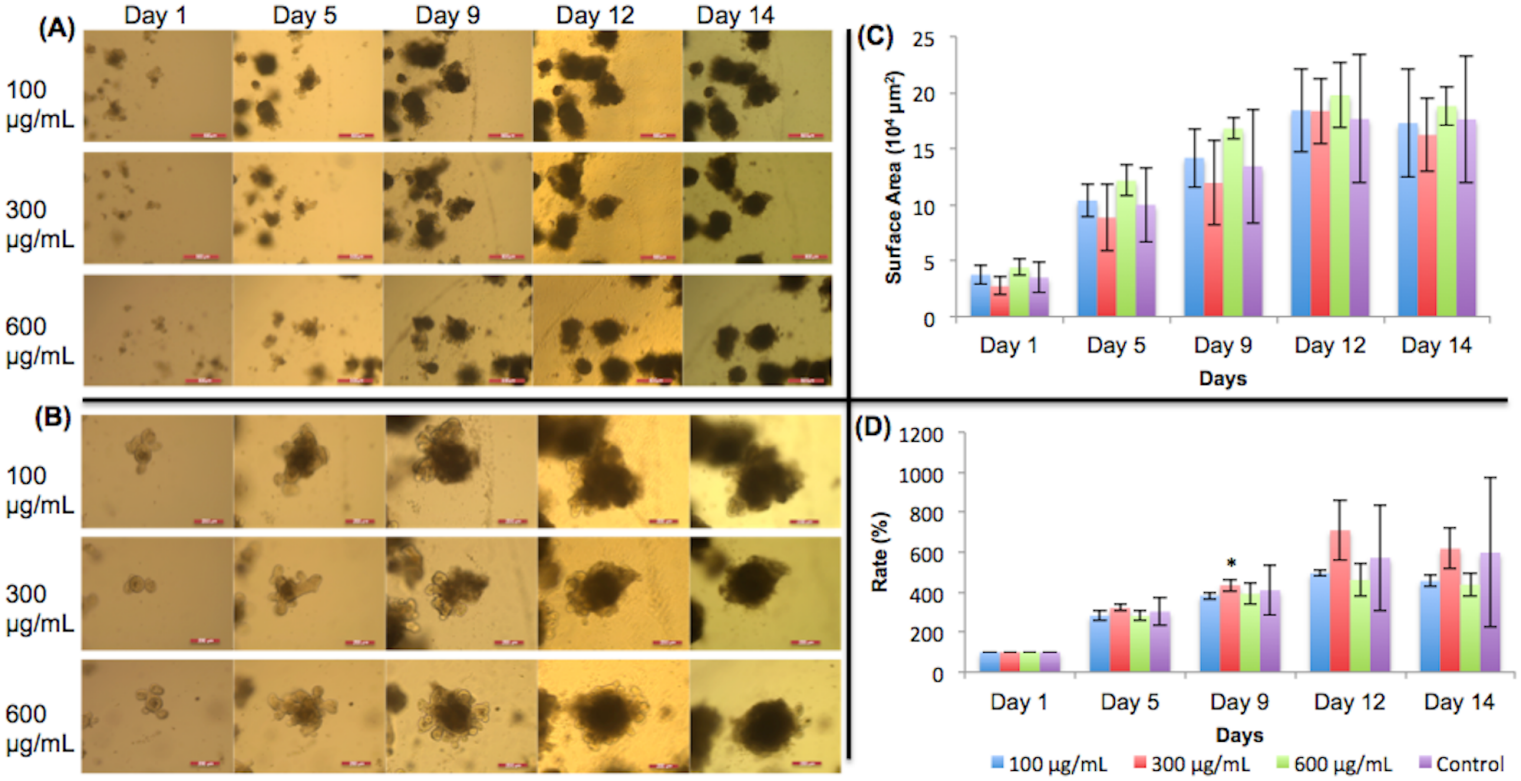
Chlorogenic acid treated organoid growth patterns. The surface areas of three organoids were measured for each concentration in each day. (A) 5X magnification of organoids given chlorogenic acid. Day 9 –day 14 show organoids with added dietary constituent. Scale bar: 500 μm. (B) 10X magnification. Day 9 –day 14 show organoids with added dietary constituent. Scale bar: 200 μm. (C) Surface area of organoids. (D) Surface area expansion rate of organoids. There was a significant change in growth on Day 9 for 300 μg/mL organoids. However, this change was not observed after Day 9 in any of the concentrations. Significance values were calculated using unpaired, two-tailed Student’s t-test with n = 3. *p<0.05.

Fig 5A and 5B show the 5X and 10X magnification, respectively, of the organoids given different concentrations of chlorogenic acid. Fig 5d shows similar growth rates from day 1 to day 5. On day 9, 300 μg/mL of chlorogenic acid organoids grew significantly more than the control. However, on days 12 and 14, this change was not reproduced in any concentration. As seen in Fig 5C and 5D, the surface areas and growth rates did not change much between day 12 and day 14, but this was not unusual because other food nutrient models exhibited the same pattern.

### Caffeic acid

**Fig 6:**
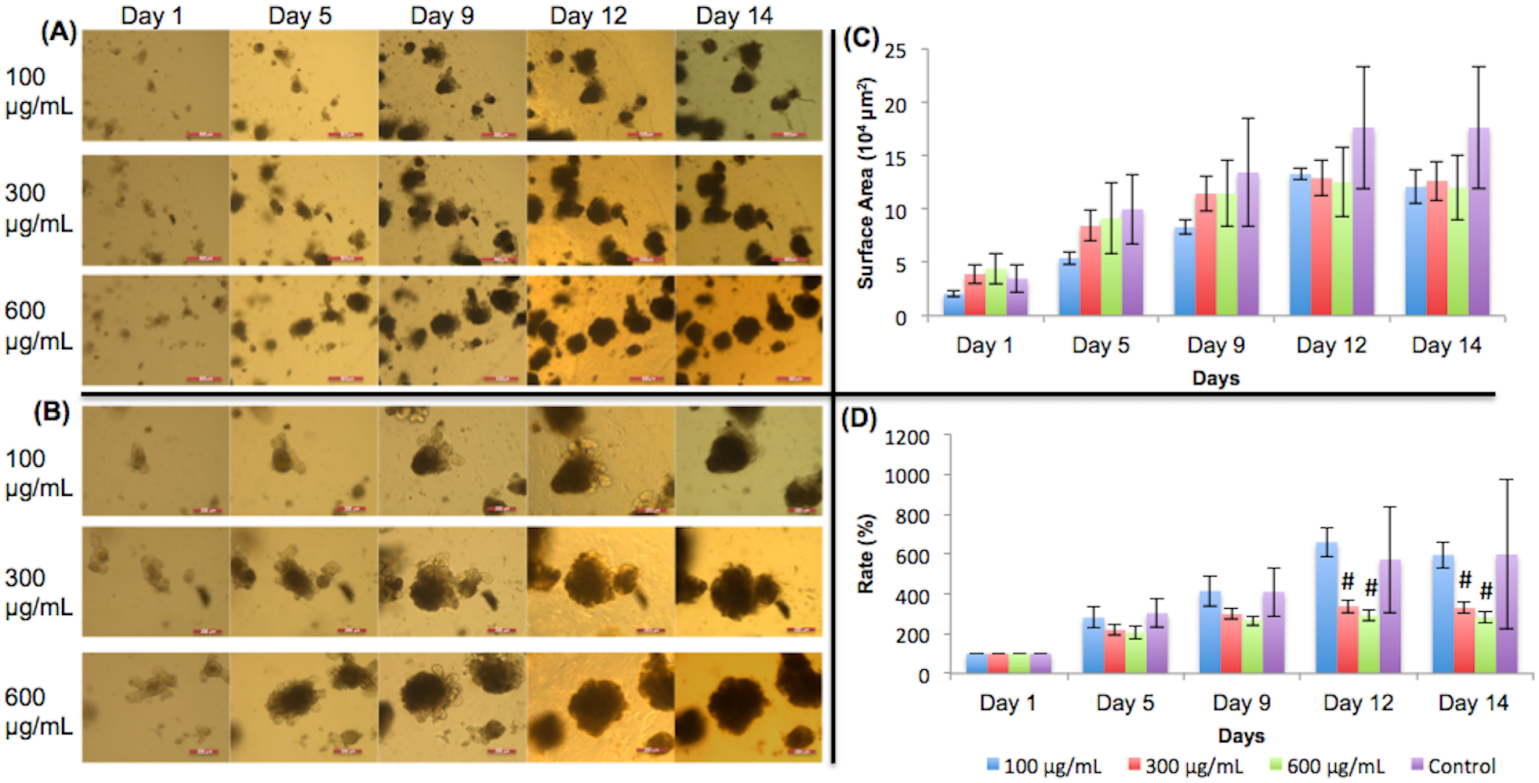
Caffeic acid treated organoid growth patterns. (A) 5X magnification of organoids given caffeic acid. Day 9 –day 14 show organoids with added dietary constituent. Scale bar: 500 μm. (B) 10X magnification. Day 9 –day 14 show organoids with added dietary constituent. Scale bar: 200 μm. (C) Surface area of organoids. (D) Surface area expansion rate of organoids. Organoid growths were inhibited significantly by 300 and 600 μg/mL caffeic acid on days 12 and day 14 when compared to the 100 μg/mL organoids. These results are similar to those of Prasad et al. (2011), where caffeic acid’s effects on cancerous cells were studied. Caffeic acid may have inhibited growth by increasing the cytotoxicity in the environment. Significance values were calculated using unpaired, two-tailed Student’s t-test with n = 3. *p<0.05. #p value < 0.05 when 300 and 600 μg/mL were compared to 100 μg/mL in a t-test.

In Fig 6D, growth rates on days 1 and day 5 were similar, which is to be expected. Starting on day 9, organoids treated with 100 μg/mL of caffeic acid exhibited faster growth than organoids treated with other concentrations. The organoids treated with 100 μg/mL of caffeic acid grew similarly to the control; thus, growth of these organoids was not inhibited. However, data from day 9 forward showed that higher concentrations of 300 μg/mL and 600 μg/mL were associated with inhibited growth as compared to 100 μg/mL caffeic acid. This is visually shown in Fig 6B. Crypts can still be observed in the organoids given 100 μg/mL of caffeic acid at day 12 and day 14. In the organoids treated with 300 μg/mL, fewer crypts can be seen than in the 100 μg/mL concentration. In the organoids treated with 600 μg/mL, the least number of crypts are observed on day 12 and day 14. Because there are stem cells located in the crypts, these organoids are expected to grow less if fewer crypts are observed.

### Curcumin

**Fig 7:**
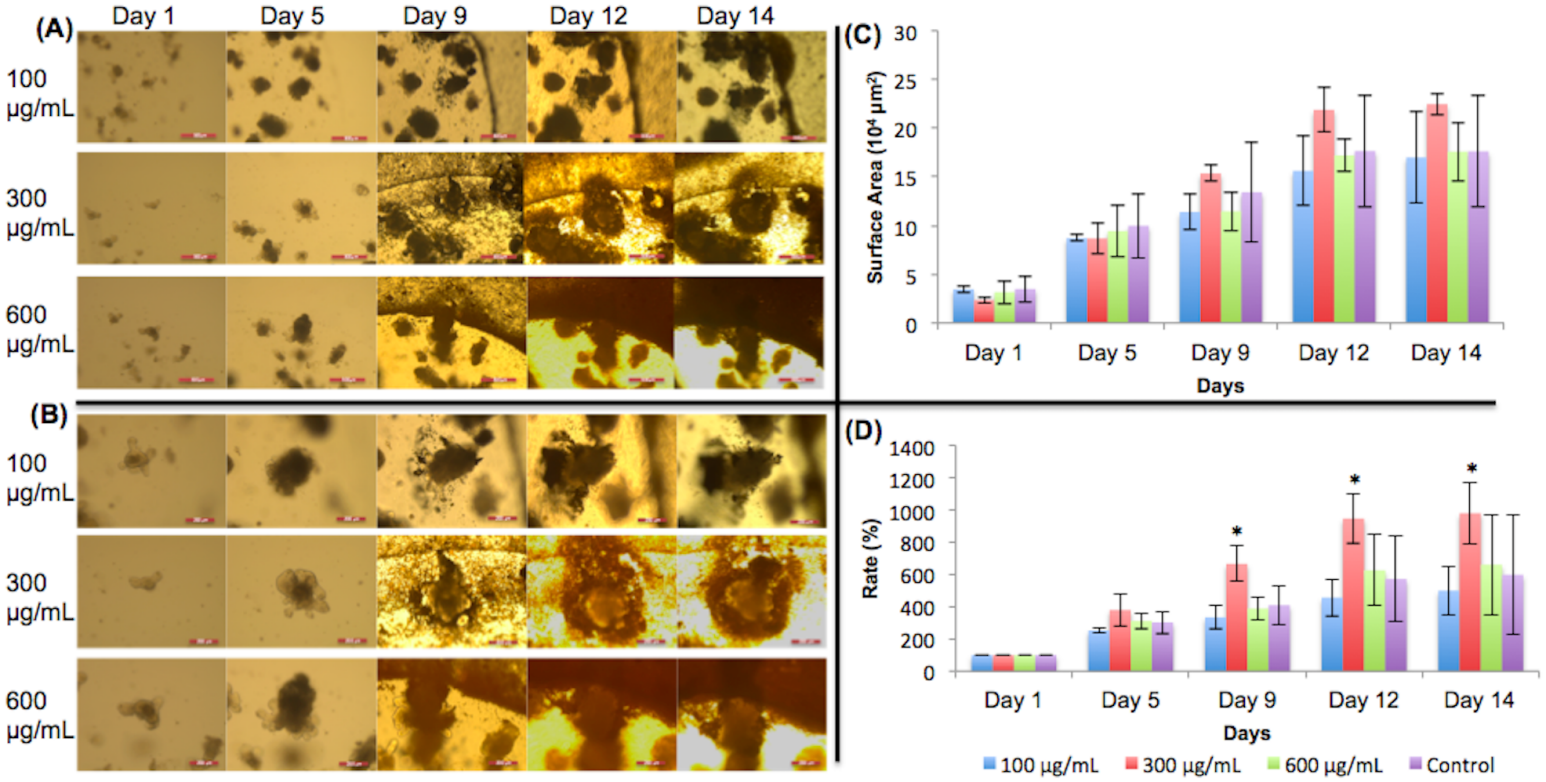
Curcumin treated organoid growth patterns. (A) 5X magnification of organoids given curcumin. Day 9 –day 14 show organoids with added curcumin. Scale bar: 500 μm. (B) 10X magnification. Day 9 –day 14 show organoids with added curcumin. Scale bar: 200 μm. (C) Surface area of organoids. (D) Surface area expansion rate of organoids. On days 9, 12, and 14, 300 μg/mL curcumin-treated organoids grew significantly faster than the control. Significance values were calculated using unpaired, two-tailed Student’s t-test with n = 3. *p<0.05.

Fig 7D shows that the organoids grew significantly faster following treatment with 300 μg/mL curcumin treatment versus the other treatment concentrations (p < 0.05). Fig 7B demonstrates that from day 9 forward, the organoids treated with 300 μg/mL were visually larger than the other organoids. Treatment with the other two concentrations did not have noticeable effects on organoid growth rate, which was similar to that in the control. From the pictures and graphs, it can be observed that curcumin influences organoid targets within a certain concentration.

**Fig 8:**
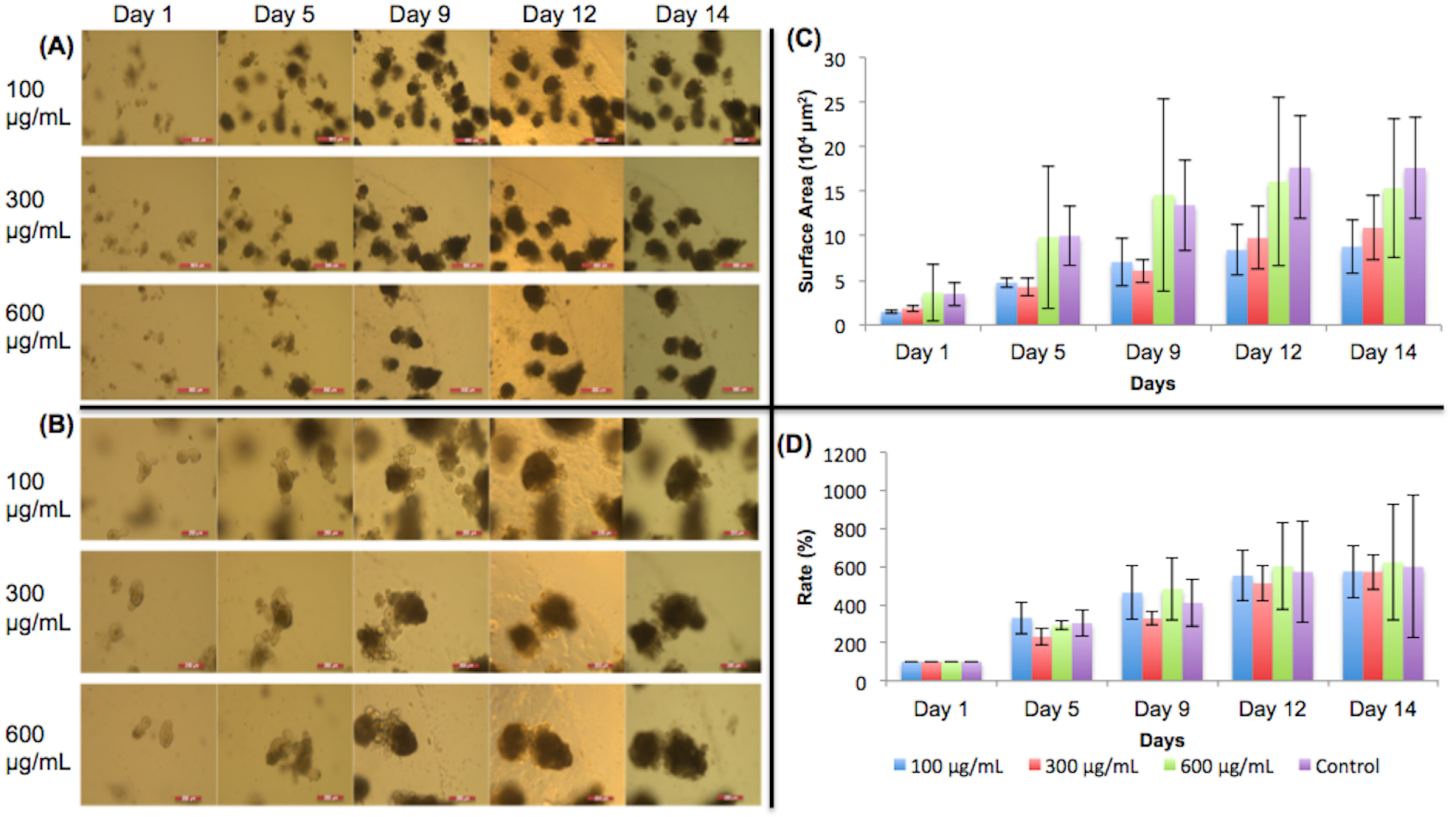
Ethanol treated organoid growth patterns. (A) 5X magnification of organoids given ethanol and their growth progressions. Day 9 –day 14 show organoids with added dietary constituent. Scale bar: 500 μm. (B) 10X magnification. Day 9 –day 14 show organoids with added dietary constituent. Scale bar: 200 μm. (C) Surface area of organoids with standard deviations. (D) Surface area expansion rate of organoids. The growth rates were not significantly different from the control. Significance values were calculated using unpaired, two-tailed Student’s t-test with n = 3. *p<0.05.

Because curcumin was dissolved in ethanol, one group of organoids was treated with only ethanol as a vehicle control. When ethanol was added on day 8 in Fig 8D, the growth rates among different concentrations did not differ significantly from one another over the next few days (p>0.05). The growth rates of organoids treated with different concentrations of ethanol did not significantly differ from the control rate, which had no added nutrients. Therefore, varying amounts of ethanol did not noticeably enhance or inhibit organoid growth.

### mHPP

**Fig 9:**
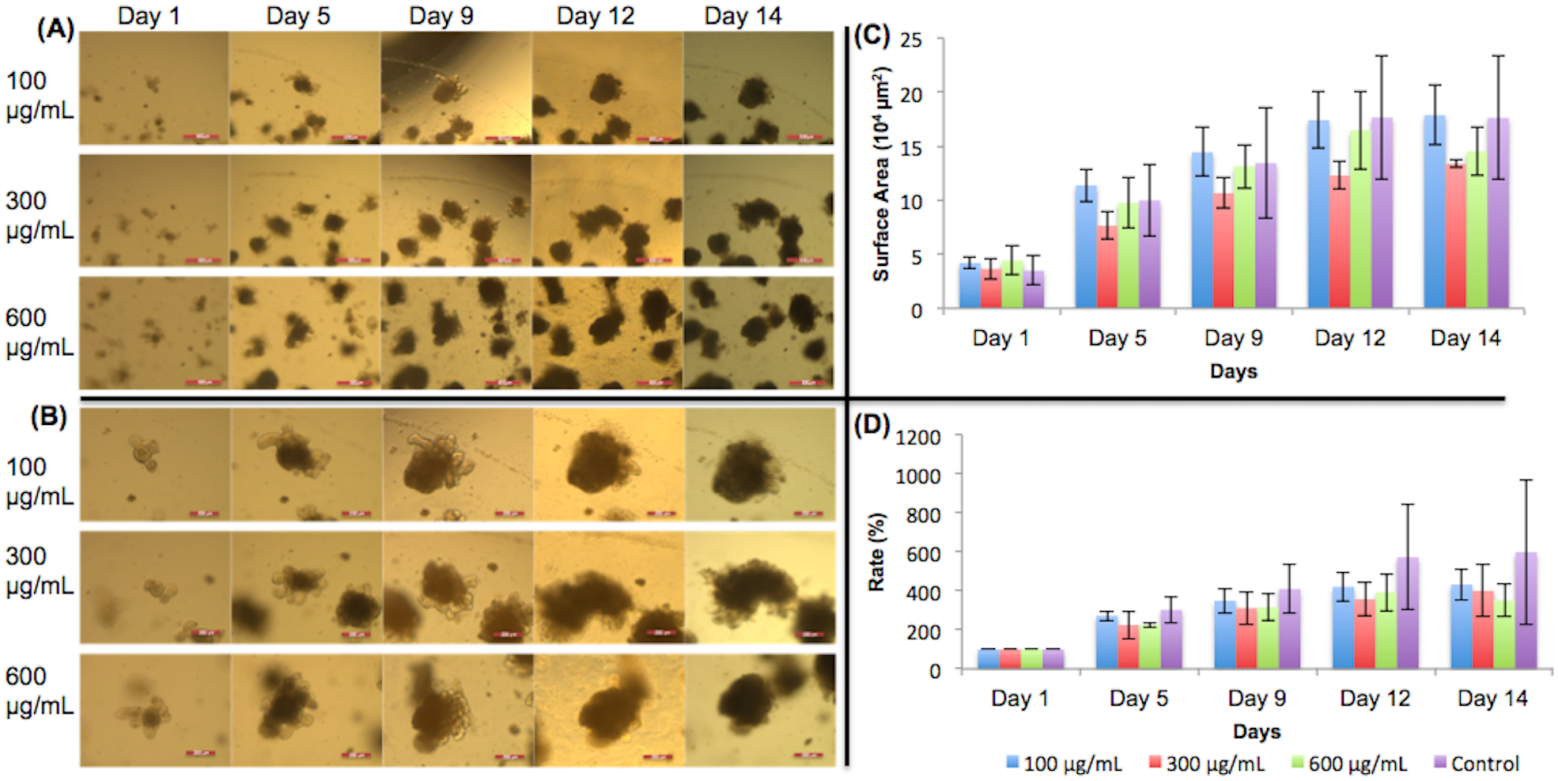
mHPP treated organoid growth patterns. (A) 5X magnification of organoids given m-hydroxyphenylpropionic acid. Day 9 –day 14 show organoids with added mHPP. Scale bar: 500 μm. (B) 10X magnification. Day 9 –day 14 show organoids with added mHPP. Scale bar: 200 μm. (C) Surface area of organoids. (D) Surface area expansion rate of organoids. mHPP may have inhibited growth slightly, but no significant growth changes compared to control. Significance values were calculated using unpaired, two-tailed Student’s t-test with n = 3. *p<0.05.

Organoids treated with mHPP showed slightly inhibited growth compared to controls. The organoid crypts can be seen shrinking slightly in Fig 9B. Because few studies have been conducted to observe the effects of mHPP on stem cells, mHPP may have positive effects on organoids in parameters other than their growth.

### Control (no dietary nutrient added)

**Fig 10:**
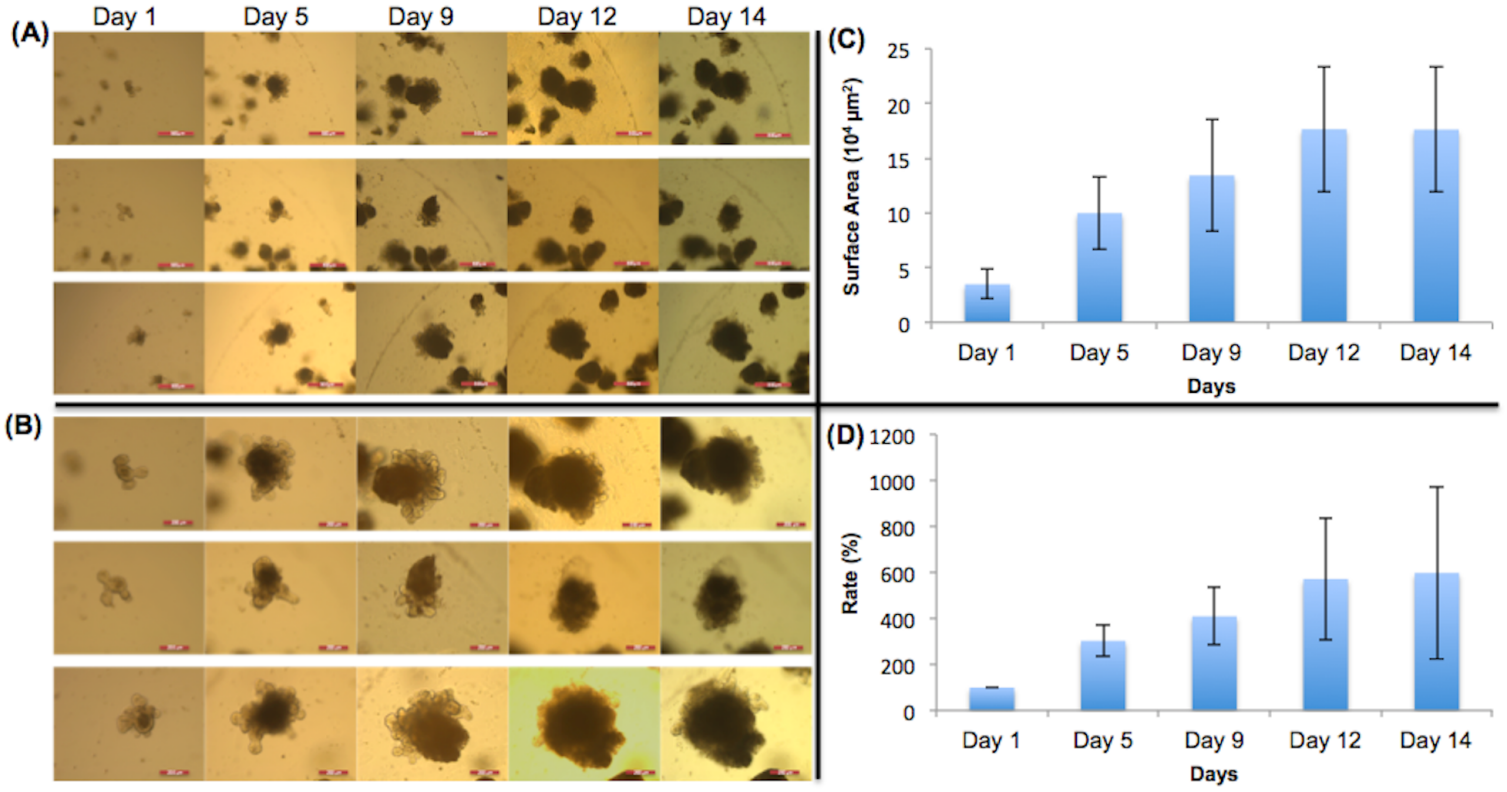
Control group of organoids. (A) 5X magnification of organoids given no dietary constituents at all during the experiment. Scale bar: 500 μm. (B) 10X magnification. No dietary constituents added during the duration of the experiment. Scale bar: 200 μm. (C) Surface area of organoids. (D) Surface area expansion rate of organoids. The organoids crypts proliferated, expanded, and became denser, increasing the sizes of the organoids.

No nutrients were added to the control (Fig 10). The graphs show that organoid growth increased until day 12, and then did not grow much further between day 12 and day 14. The images of different magnifications show that the organoids grew continuously throughout the experimental period. The proliferation and differentiation of stem cells in the crypts increased the size and density of the organoids.

## Discussion

In our study, organoid growth was only affected on day 12 with 300 μg/mL MSG, and the rest were not affected by the treatment. This leads to the conclusion that MSG did not affect intestinal organoid growth. However, the effects of MSG observed in this study did not correlate with the results of previous studies. Earlier reports indicated that MSG should inhibit cell growth [14]. Previously, Feng et al. [14] reported that MSG inhibited the growth of crypts derived from porcine intestines. We hypothesized that mouse intestinal organoids would respond to MSG treatment in a similar manner. Because our results were dissimilar to other results, the effects of MSG on organoid growth using our methods should be evaluated further.

Organoids treated with vitamin C did not show a growth pattern significantly different from that observed for the controls. While vitamin C is essential for life and provides multiple health benefits [15–17], the results of this study suggest that higher doses of vitamin C are unnecessary and inefficient in affecting organoid growth patterns [25]. MacDonald et al. [25] also studied the effect of different doses of vitamin C on the performance of the vitamin C transporter SVCT1 in Caco-2 cells. They observed that SCVT1 expression in this cell line did not change significantly in lower concentrations of vitamin C (45 and 450 μg/mL) but decreased in the presence of high levels of vitamin C (4.5 mg/mL).

Our results show that 300 μg/mL chlorogenic acid increased organoid growth compared to the control on day 9. However, other than this observation on day 9, there were no significant effects attributable to chlorogenic acid treatment. This leads to the conclusion that chlorogenic acid did not have any significant effects on organoid growth. In a previous study, Duda-Chodak et al. [26] observed significant effects of chlorogenic acid, along with quercetin and epigallocatechin, on the growth of Caco-2 cells. They reported that chlorogenic acid stimulated cell growth in a time- and concentration-dependent manner. Since our results are dissimilar to theirs, the effects of chlorogenic acid on the growth of murine intestinal organoids using our methods should be evaluated further.

Intestinal organoids treated with 100 μg/mL of caffeic acid grew similarly to the control organoids. However, higher concentrations of caffeic acid significantly inhibited organoid growth. While few studies have been conducted on the effects of caffeic acid on stem cell growth, there have been other studies showing a relationship between caffeic acid and cancer cell growth. Prasad et al. [13] reported that caffeic acid inhibited fibrosarcoma cell line in vitro in a concentration-dependent manner using a Dulbecco’s Modified Eagle’s Medium (DMEM) culture system. Higher caffeic acid concentrations exhibited increased cytotoxic effects on HT-1080 cells. While the cells studied were HT-1080 fibrosarcoma cell lines, higher concentrations of caffeic acid may have affected the intestinal organoids in a similar way by increasing cytotoxicity and inhibiting the proliferation of crypts. Additionally, this demonstrates that organoids may serve as another tool other than cell culture systems to evaluate toxicity.

Results showed that 300 μg/mL of curcumin significantly stimulated organoid growth versus other concentrations. This suggests that there is an optimal concentration range for curcumin usage. Doses too low or too high will not have a significant effect on organoid growth. This also has potential implications in cancer treatment. Previous studies have demonstrated the inhibitory effects of curcumin on different types of cancers, such as Dhandapani et al. [20] and Fong et al. [27]. However, it has been observed that curcumin does not harm normal cells. Thus, there is potential for curcumin to be used to inhibit cancer cell growth while stimulating normal cell proliferation.

Ethanol did not significantly influence organoid growth as curcumin did. A group of organoids was given ethanol as a vehicle control because curcumin was dissolved in ethanol. Because ethanol alone did not alter intestinal organoid growth rate, it can be concluded that only curcumin exhibited an effect on the organoids. A related study observed the effects of alcohol consumption on ISC dysfunction and transformation [28]. Their results reported ISC dysregulation from both chronic and acute exposures of alcohol [28]. In another related study, it is reported that alcohol increased the miR-212 expression in intestinal epithelial cells [29].

Our results show that mHPP did not significantly affect intestinal organoid growth. Because there are few studies to compare our results to, our findings may not be definitive and conclusive. We conclude that it is possible that mHPP may have an effect on cells in different ways other than by influencing growth rate. Caffeic acid is a polyphenol [22], and polyphenols are known to exert antioxidant properties [30]. Because mHPP is a metabolite of caffeic acid, mHPP may benefit organoids by eliminating oxidative stress [22]. Further studies are needed to confirm this speculation.

Because the dietary constituents exhibited different saturation abilities and were dissolved with different methods, this could have created some experimental bias. Additionally, three organoids were chosen from each well to serve as representatives of the organoid population in that well. This sample size is small, and the conclusions could potentially be more definitive if the sample size (power) were increased.

## Supporting information

S1 Table Methods for dissolving the various constituents used in this study. (TIF)
