# Supplementary figures and images for: Effects of six common dietary nutrients on murine intestinal organoid growth
Source: PLoS One. 2018 Feb 1;13(2):e0191517. doi: 10.1371/journal.pone.0191517 (PMC5794098; doi:10.1371/journal.pone.0191517)

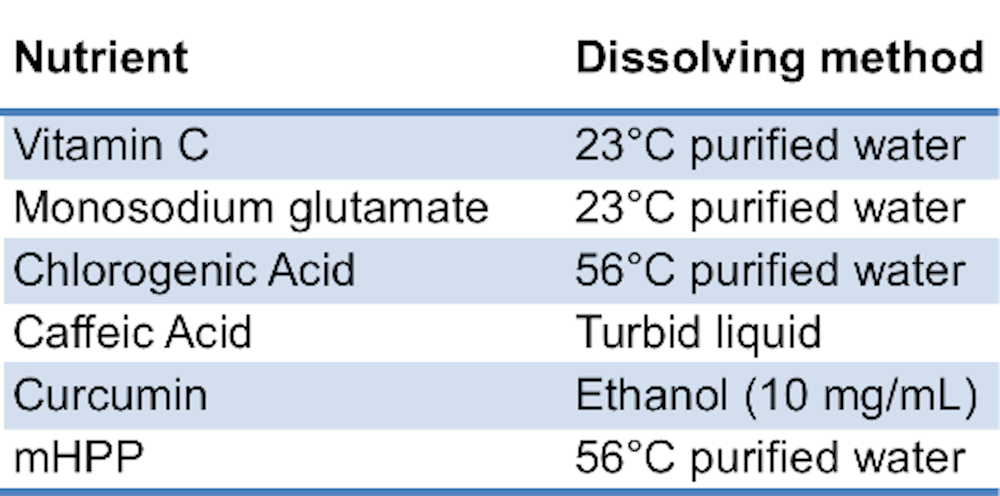

Supplement: S1 Table — (TIF) [file pone.0191517.s001.tif]
